# Integrated characterization of aroma, taste, and sensory properties of four commercial mutton soups from different regions of China

**DOI:** 10.1016/j.fochx.2026.104559

**Published:** 2026-10-01

**Authors:** Can Xiang, Xue Dai, Zhaojun Chen, Chunjiang Zhang, Dequan Zhang, Sining Li, Caiyan Huang

**Affiliations:** a Institute of Urban Agriculture, Chinese Academy of Agricultural Sciences, Chengdu National Agricultural Science and Technology Center, Chengdu 610213, China; b Department of Flavor Chemistry (150h), Institute of Food Science and Biotechnology, University of Hohenheim, Fruwirthstraße 12, 70599 Stuttgart, Germany; c College of Pharmacy and Food, Southwest Minzu University, Chengdu 610041, China; d Institute of Food Science and Technology, Chinese Academy of Agriculture Sciences, Beijing 100193, China; e Guizhou Provincial Academy of Agricultural Sciences, Guiyang 550000, China

**Keywords:** Mutton soup, Aroma profile, HS-GC-IMS, Taste-active compound, Sensory evaluation

## Abstract

Mutton soups from different regions of China exhibit distinct flavor characteristics, but the chemical basis of these differences remains insufficiently understood. Four commercial products from different regions in China were compared using integrated instrumental, chemical, and sensory analyses. Among 77 detected volatile compounds, 22 had estimated odor activity values greater than 1. Five lipid oxidation-associated compounds were common to all products, whereas Guoshi contained relatively high levels of several spice-associated volatiles and showed the most distinct headspace fingerprint. Multivariate analysis identified ten potential discriminant volatile compounds. Jianyang had the highest total free amino acid and 5′-nucleotide contents, reaching 2125.44 and 66.89 mg/L, respectively. It also showed the highest equivalent umami concentration (5.802 g MSG/100 mL) and received the highest taste, aroma, and overall acceptability scores. These results provide a comparative basis for understanding flavor variation among commercial mutton soups and may support their quality evaluation and flavor optimization.

## Highlights

• Guoshi exhibited the most distinctive volatile fingerprint among all samples.
• Lipid oxidation-associated volatiles were shared across all four samples.
• Spice-associated volatiles contributed to product discrimination.
• Jianyang received the highest taste, aroma, and overall acceptability scores.

## 1 Introduction

Mutton soup is a traditional meat product widely consumed in China and is appreciated for its distinctive flavor and nutritional value (An et al., 2023). Owing to China's vast geographical diversity, considerable differences exist in dietary habits, culinary traditions, ingredient selection, and cooking practices among regions, leading to the development of a wide variety of mutton soups with distinct regional characteristics (Lee et al., 2021). Among the numerous regional styles of mutton soup, those originating from Inner Mongolia, Shanxi, Sichuan, and Shandong province are well known for their unique preparation methods and flavor characteristics. Differences in sheep breeds, ingredient selection, seasoning formulations, and processing practices may result in substantial variation in their flavor profiles. With the increasing commercialization of traditional foods, maintaining regional flavor characteristics while ensuring product consistency has become an important challenge (Haider et al., 2024). Therefore, characterizing the flavor profiles of mutton soups prepared in different regions and identifying the compounds responsible for their sensory properties are important for quality control, flavor regulation, and product development.

Flavor is a major determinant of food quality and consumer acceptance and is generally perceived through the combined sensations of aroma and taste (Huang et al., 2023). Aroma is primarily determined by volatile compounds generated during cooking through lipid oxidation, Maillard reactions, and the release of spice-derived volatiles (Li et al., 2021; Madruga et al., 2010). Lipid oxidation products, including aldehydes, ketones, alcohols, acids, and esters, contribute characteristic fatty, grassy, fruity, and roasted notes to meat products (Argemí I Armengol et al., 2019; Sun et al., 2023). In addition, spices commonly used in regional mutton soups introduce volatile compounds such as terpenes and aromatic aldehydes, further increasing aroma complexity and regional distinctiveness. Consequently, variations in raw materials, processing conditions, and seasoning formulations may lead to distinct aroma profiles among mutton soups prepared in different regions.

Taste is another essential component of flavor perception and is mainly influenced by non-volatile compounds. Free amino acids and nucleotides are responsible for the taste properties of meat broths (Jeong et al., 2020). Among them, glutamic acid and aspartic acid are important umami amino acids, whereas glycine and alanine contribute sweetness and leucine, valine, and phenylalanine are associated with bitterness (Lai et al., 2022). Furthermore, nucleotides such as inosine 5′-monophosphate (IMP), guanosine 5′-monophosphate (GMP), and adenosine 5′-monophosphate (AMP) can interact synergistically with umami amino acids to enhance umami intensity (Ramesh et al., 2025). Variations in the concentrations of these taste-active compounds may therefore contribute to differences in taste among the mutton soups from different regions.

Despite the importance of both aroma and taste to the overall flavor of mutton soup, these two aspects have generally been examined separately (Huang et al., 2022; Zhang et al., 2025). Previous studies have mainly focused on either volatile compounds or non-volatile taste components, while relatively few have combined complementary volatile profiling techniques, electronic sensing, quantitative analysis of taste-active compounds, and sensory evaluation. It therefore remains unclear which flavor compounds are shared among mutton soups prepared in different regions and which compounds are associated with differences among individual products. Moreover, to our knowledge, commercial mutton soups representing the Baerhu of Inner Mongolia province, the Guoshi of Shanxi province, the Jianyang of Sichuan province, and the Shanxian of Shandong province have not been compared within the same analytical framework. We hypothesized that these products would share a group of flavor compounds related to their common meat-based matrix but would differ in the composition and relative abundance of volatile and non-volatile compounds, resulting in distinguishable flavor profiles.

Accordingly, this study compared four commercial mutton soups named Baerhu, Guoshi, Jianyang, and Shanxian mutton soup. The main objective was to characterize their shared and differential flavor components and to identify compounds associated with product discrimination. Aroma profiles were examined using an electronic nose (E-nose), gas chromatography-ion mobility spectrometry (GC-IMS), and gas chromatography–mass spectrometry (GC–MS). Taste characteristics were evaluated using an electronic tongue (E-tongue) together with quantitative analysis of free amino acids and nucleotides. Sensory evaluation was conducted to assess taste, aroma, oiliness, color, and overall acceptability. Principal component analysis (PCA) and partial least squares discriminant analysis (PLS–DA) were used to compare the four products and screen potential discriminant compounds. This study focused on compositional differences among the selected commercial products rather than determining the effects of individual factors such as sheep breed, seasoning composition, or processing conditions. The results provide information on the chemical components associated with their flavor differences and may support flavor regulation and product development.

## 2 Materials and methods

### 2.1 Reagents

5′-GMP (98%), 5′-IMP (≥98%), 5′-AMP (≥99%), and 2,4,6-trimethylpyridine (99%) were obtained from Sigma-Aldrich Trading Co., Ltd. (Shanghai, China). Perchloric acid (HClO_4_), sodium hydroxide (NaOH), and potassium dihydrogen phosphate (KH_2_PO_4_) were purchased from Sinopharm Chemical Reagent Co., Ltd. (Shanghai, China). 5-Sulfosalicylic acid was obtained from Beijing Solarbio Science & Technology Co., Ltd. (Beijing, China). Methanol (HPLC grade) was purchased from Macklin Biochemical Co., Ltd. (Shanghai, China). Unless otherwise specified, all chemicals and reagents used in this study were of analytical grade.

### 2.2 Sample information and preparation

Four commercially mutton soup products from different regions in China were selected, namely Shanxian mutton soup from Shandong province, Baerhu mutton soup from Inner Mongolia province, Guoshi mutton soup from Shanxi province, and Jianyang mutton soup from Sichuan province. Shanxian mutton soup was produced by Shandong Baishoufang Food Co., Ltd. (Heze, Shandong, China) on July 1, 2025. It was prepared from Jining Grey goat raised in Heze, and its declared ingredients were water, bone-in goat meat, salt, and spices. Baerhu mutton soup was produced by Hulunbuir Baerhu Animal Husbandry Development Co., Ltd. (Hulunbuir, Inner Mongolia, China) on April 28, 2025. It contained Baerhu mutton, drinking water, salt, goji berries, and spices. Guoshi mutton soup was produced by Shanxi Guoshi Food Industry Co., Ltd. (Huguan County, Changzhi, Shanxi, China) on May 18, 2025. It was prepared from locally sourced mutton sheep offal, with drinking water, salt, and spices. The three packaged products were purchased from online platform and stored unopened at room temperature according to the instructions on their labels. Jianyang mutton soup was freshly prepared by Jianyang Tiandi Sheep Food Co., Ltd. (Jianyang, Sichuan, China), using Jianzhou big-eared goat, and was collected directly at the production site. The individual spices and their proportions were not disclosed for products whose labels listed only “spices.”

Before opening, the packaged samples were stored at room temperature according to the manufacturers' instructions. The entire contents of each package were transferred into a microwave-safe glass container and heated at 800 W for 3 min using a microwave. After cooling to room temperature, the samples were passed through a stainless-steel sieve to remove meat, bones, and other solid ingredients. The resulting broth was mixed thoroughly, divided into aliquots, and stored at −80 °C until analysis. Before each analysis, the samples were thawed at room temperature and mixed thoroughly. Each package was prepared independently, and repeated freeze–thaw cycles were avoided.

### 2.3 E-nose analysis

E-nose analysis was performed using an E-nose system (cNose, Shanghai Bosin Industrial Development Co., Ltd., Shanghai, China) equipped with 14 metal oxide sensors (Table S1) according to the previously described method (Mouro et al., 2023). The sensors differed in their nominal response ranges. S1, S7–S9, S13, and S14 were mainly sensitive to alkanes, methane, natural gas, biogas, petroleum ether vapors, and other combustible gases. S2, S6, and S12 responded primarily to alcohols, aldehydes, ketones, short-chain alkanes, anisole, and other organic or aromatic compounds. S3–S5, S10, and S11 were mainly sensitive to ozone, sulfur compounds including hydrogen sulfide, nitrogen-containing compounds including ammonia, hydrogen, and allyl sulfides, respectively. For analysis, 2 mL of each mutton soup sample was placed into a 20 mL vial and equilibrated at 50 °C for 30 min (Liu et al., 2021). After the sample cooled to 25 °C, the E-nose sensor was inserted into the samples to analyze the headspace volatiles. For each product, three separately prepared sample replicates were analyzed. Each replicate was measured three times under identical conditions, and the three instrumental readings were averaged before statistical analysis.

### 2.4 E-tongue analysis

Taste profiles were analyzed using an electronic tongue system (SA402B, INSENT, Japan) according to the previously described method (Xu et al., 2023). Briefly, each sample was diluted fivefold with ultrapure water and filtered through medium-speed filter paper. The filtrate was transferred to the sample cup for analysis, and data was collected for 120 s. The responses of the taste sensors were recorded and used for subsequent statistical analysis. All measurements were performed in triplicate.

### 2.5 Determination of volatile compounds

#### 2.5.1 GC–MS analysis

Volatile compounds were analyzed by headspace solid-phase microextraction (HS-SPME) coupled with GC–MS according to a previously reported method with minor modifications (Zhou et al., 2024). An Agilent 7890B gas chromatograph coupled with a 5977A mass selective detector (Agilent Technologies, USA) was used. Volatiles were extracted using a 50/30 μm thickness divinylbenzene/carboxen/polydimethylsiloxane (DVB/CAR/PDMS) fiber (Supelco, USA). Before its first use, the fiber was conditioned at 250 °C for 30 min. Before each batch of samples, it was desorbed at 250 °C for 5 min to minimize carryover. For extraction, 6 mL of sample and 4 μg of 2,4,6-trimethylpyridine (internal standard) were added to a headspace vial. The sample was equilibrated at 60 °C for 15 min and subsequently extracted from the headspace using the DVB/CAR/PDMS fiber at 60 °C for 30 min. After extraction, the fiber was inserted into the GC inlet and thermally desorbed at 250 °C for 3 min in splitless mode. Chromatographic separation was performed on a DB-WAX capillary column (30 m × 0.25 mm × 0.25 μm, Agilent Technologie, USA). High-purity helium (≥99.999%) was used as the carrier gas at a constant flow rate of 1.0 mL/min. The oven temperature was initially maintained at 40 °C for 3 min, increased to 70 °C at 3 °C/min and held for 3 min, then increased to 180 °C at 5 °C/min and held for 3 min, and finally increased to 250 °C at 10 °C/min and held for 5 min. The total run time was 53 min. Mass spectra were acquired under electron ionization at 70 eV in full-scan mode over an *m/z* range of 33–330. Volatile compounds were tentatively identified by comparing their mass spectra with those in the NIST mass spectral library. The concentration of each flavor compound was estimated using the internal-standard method, assuming a relative response factor of 1:

$$C_{i}=\frac{A_{i}\times m_{\text{IS}}}{A_{\text{IS}}\times V_{s}}$$

where $C_{i}$ is the estimated concentration of compound i (μg/L); $A_{i}$ and $A_{\mathrm{IS}}$ are the peak areas of compound i and the internal standard, respectively; $m_{\mathrm{IS}}$ is the amount of internal standard added (μg) and $V_{S}$ is the volume of the soup sample (L). A procedural blank containing the internal-standard solution but no soup sample was subjected to the same HS-SPME-GC–MS procedure. Peaks detected in the blank were treated as background signals and excluded from the sample data.

#### 2.5.2 GC-IMS analysis

The volatile components in the mutton soup were further analyzed using GC-IMS (FlavourSpec®, G.A.S., Germany) according to a previously reported method with minor modifications (Sun et al., 2024). Briefly, 5 mL of the sample was transferred to a 20 mL headspace vial and incubated at 60 °C for 20 min. Next, 500 μL headspace gas was automatically injected into the injector, with a needle temperature of 85 °C. High-purity nitrogen served as carrier and drift gas. Separation was carried out on an MXT-WAX column (15 m × 0.53 mm × 1 μm, Restek, USA) at 60 °C. Volatile compounds were identified by comparing their retention indices (calculated using a series of n-ketones, C4–C9, as external references) and drift times with those in the NIST and GC-IMS databases.

#### 2.5.3 Calculation of odor activity values (OAVs)

OAVs were calculated to estimate the potential contribution of individual volatile compounds to the aroma profile (Ping et al., 2024), using the following equation:

$${\mathrm{OAV}}_{i}=\frac{C_{i}}{T_{i}}$$

where $C_{i}$ is the estimated concentration of volatile compound i in the mutton soup (μg/L), and $T_{i}$ is its odor threshold in water (μg/L). Odor thresholds were obtained from the literature and are listed in Table 1. Compounds with OAVs greater than 1 were considered potentially odor-active. Because the concentrations were estimated using a single internal standard with an assumed relative response factor of 1, the calculated OAVs were used for comparative screening rather than as definitive measures of sensory contribution.

**Table 1:** Volatile compounds with OAVs greater than 1 in four commercial mutton soup products.

No.	Volatile compounds	Odor threshold(μg/L)	OAV				Relative contribution (%)			
			Baerhu	Guoshi	Jianyang	Shanxian	Baerhu	Guoshi	Jianyang	Shanxian
1	Octanal	6.9	11.77	3.91	13.94	6.81	3.79	1.01	2.44	5.06
2	Valeraldehyde	12	–	1.19	1.14	1.38	–	0.31	0.20	1.03
3	Nonanal	1	183.46	111.98	200.87	73.23	59.11	28.94	35.13	54.42
4	Hexanal	4.5	–	9.98	21.78	9.64	–	2.58	3.81	7.16
5	Heptanal	3	27.92	8.91	27.51	28.61	9.00	2.30	4.81	21.26
6	Cuminaldehyde	43	–	4.52	–	–	–	1.17	–	–
7	Benzaldehyde	24	4.30	1.30	–	–	1.39	0.34	–	–
8	(*E*)-2-Octenal	3	4.28	3.81	11.81	1.58	1.38	0.98	2.07	1.17
9	2-Undecenal	0.78	–	–	24.24	4.82	–	–	4.24	3.58
10	1-Hepten-3-one	0.04	–	–	178.50	–	–	–	31.22	–
11	2-Heptanone	3	2.40	–	–	–	0.77	–	–	–
12	2-Decanone	3	2.02	1.92	–	1.69	0.65	0.50	–	1.26
13	2-Nonanone	5	1.70	–	–	1.29	0.55	–	–	0.96
14	3-Methyl-2-heptanone	8.1	2.35	–	–	–	0.76	–	–	–
15	3-Penten-2-one	1.5	28.99	–	–	–	9.34	–	–	–
16	n-Octanol	77.5	–	–	1.12	–	–	–	0.20	–
17	Linalool	7.4	6.71	29.75	8.62	–	2.16	7.69	1.51	–
18	2-Bornanol	2.4	–	–	4.91	–	–	–	0.86	–
19	1-Octen-3-ol	1.5	25.36	6.31	15.56	5.52	8.17	1.63	2.72	4.10
20	d-Limonene	500	–	1.55	–	–	–	0.40	–	–
21	Phellandrene	40	–	6.81	–	–	–	1.76	–	–
22	β-Myrcene	1.2	9.11	194.95	61.75	–	2.94	50.39	10.80	–

–: OAV < 1.

### 2.6 Determination of non-volatile compounds

#### 2.6.1 Free amino acids (FAAs)

FAAs were determined according to the method described by Wu et al. (2003). Each mutton soup was diluted threefold with ultrapure water and filtered through medium-speed filter paper. A 5 mL aliquot of the filtrate was mixed with an equal volume of 5% 5-sulfosalicylic acid solution and centrifuged at 10,000 ×*g* for 10 min at 4 °C. The pH of the resulting supernatant was adjusted to 2.0, followed by filtration through a 0.22 μm nylon membrane. The filtrate was then analyzed using an amino acid analyzer (A300, Amino, Germany).

#### 2.6.2 Nucleotides

The contents of 5′-nucleotides were determined according to a previously reported method with slight modifications (Qi et al., 2017). Briefly, 5 mL mutton soup was mixed with 2 mL 5% HClO_4_ and centrifuged (10,000 r min^−1^) at 4 °C for 10 min. Then, 5 mL supernatant was adjusted to pH 5.8 with 1.0 M NaOH solution and diluted to 10 mL with ultrapure water and filtered through a 0.22 μm membrane filter. Finally, the filtrate was analyzed by HPLC (2695, Waters Technology, USA) with a Symmetry Ultimate AQ-C18 column. The mobile phase consisted of 0.05 mol L^−1^ KH_2_PO_4_ buffer and methanol.

#### 2.6.3 Calculation of taste activity value (TAV) and equivalent umami concentration (EUC)

The TAV was calculated as the ratio of the measured concentration to the corresponding taste threshold.

The EUC was calculated according to the method described by Zhu et al. (2022). In detail, the concentrations of glutamic acid, aspartic acid, 5′-AMP, 5′-IMP, and 5′-GMP were originally determined in mg/L. Before calculating EUC, all concentrations were converted to g/100 mL by dividing the values in mg/L by 10,000. The EUC was then calculated using the following equation: using the following equation:

$$\mathrm{EUC}=\sum a_{i}b_{i}+1218\left(\sum a_{i}b_{i}\right)\left(\sum a_{j}b_{j}\right)$$

where $a_{i}$ is the concentration of each umami amino acid in the soup filtrate (g/100 mL), $b_{i}$ is its relative umami coefficient (1.00 for glutamic acid and 0.077 for aspartic acid), $a_{j}$ is the concentration of each taste-active 5′-nucleotide (g/100 mL), and $b_{j}$ is its relative umami coefficient (1.00 for 5′-IMP, 2.30 for 5′-GMP, and 0.18 for 5′-AMP). The constant 1218 represents the synergistic effect between umami amino acids and 5′-nucleotides.

### 2.7 Sensory evaluation

Sensory evaluation was conducted by a trained panel consisting of 12 assessors (6 males and 6 females, aged 20–40 years. The assessors were familiarized with the sensory characteristics of mutton soup, the definitions of the evaluated attributes, and the use of the scoring scale. The sensory procedure was developed with reference to ISO 13299:2016. The evaluated attributes included taste, aroma, oiliness, color, and overall acceptability. The attributes were selected through preliminary evaluation and discussion among the panelists based on the main sensory characteristics of mutton soup. Each attribute was evaluated independently using a 10-point scale. The definitions and scale anchors are provided in Table S2 (Yu et al., 2025). Before evaluation, the samples were maintained at approximately 45 °C, and 20 mL of each sample was served in a cup labelled with a random three-digit code. The presentation order was randomized for each assessor. A 2 min interval was scheduled between successive samples, during which purified water and unsalted crackers were used for palate cleansing. Each sample was evaluated in three separate sessions under the same conditions.

Ethical approval was not required by the Chinese Academy of Agricultural Sciences for this type of sensory study. All participants were informed of the study procedures and any potential risks before participation. Participation was voluntary, and participants were free to withdraw from the study at any time.

### 2.8 Statistical analysis

For each product, three separate unopened commercial packages were independently prepared and analyzed. Each package constituted one replicate, and the results are expressed as the mean ± standard deviation of three package-level replicates. Repeated instrumental measurements, where applicable, were not considered independent replicates. Significant difference among samples were analyzed by one-way analysis of variance (ANOVA) followed by Duncan's multiple range test using SPSS version 27.0 (IBM Corp., New York, USA). Differences were considered significant at *P* < 0.05. PCA and Pearson correlation analysis was performed using OriginPro 2025 (Originlab Corp., Northampton, USA), while PLS–DA was conducted using SIMCA 14.1 software (Umetrics, Umeå, Sweden).

## 3 Results and discussion

### 3.1 Aroma characteristics of four regional mutton soups

#### 3.1.1 Overall odor profiles and sample discrimination based on E-nose responses

To obtain a rapid overview of the odor profiles of the four mutton soups, an E-nose was employed. By simulating the human olfactory system, the E-nose enables the discrimination and classification of samples based on their volatile characteristics (Li et al., 2024). Higher sensor response values generally indicate a greater abundance of the corresponding volatile compounds, thereby reflecting differences in odor characteristics among samples (Zhang et al., 2021). As shown in Fig. 1a, sensors S1, S2, and S9 exhibited relatively high responses to all samples, suggesting that the four mutton soups shared certain common odor characteristics, mainly associated with short-chain alkanes, alcohols, and aldehydes. However, the response intensities of these sensors were generally higher in Guoshi, indicating higher levels of the corresponding volatile compounds. In addition, Guoshi showed stronger responses in sensors S4, S5, S6, and S12, which are sensitive to sulfur-containing compounds, nitrogen-containing compounds, aldehydes, aromatic compounds, ketones, and alcohols. These results suggest that the volatile composition of Guoshi differed from that of the other samples and contained a wider variety of potentially odor-active compounds. Overall, Guoshi exhibited a more distinctive odor profile than the other mutton soups.

**Fig. 1:**
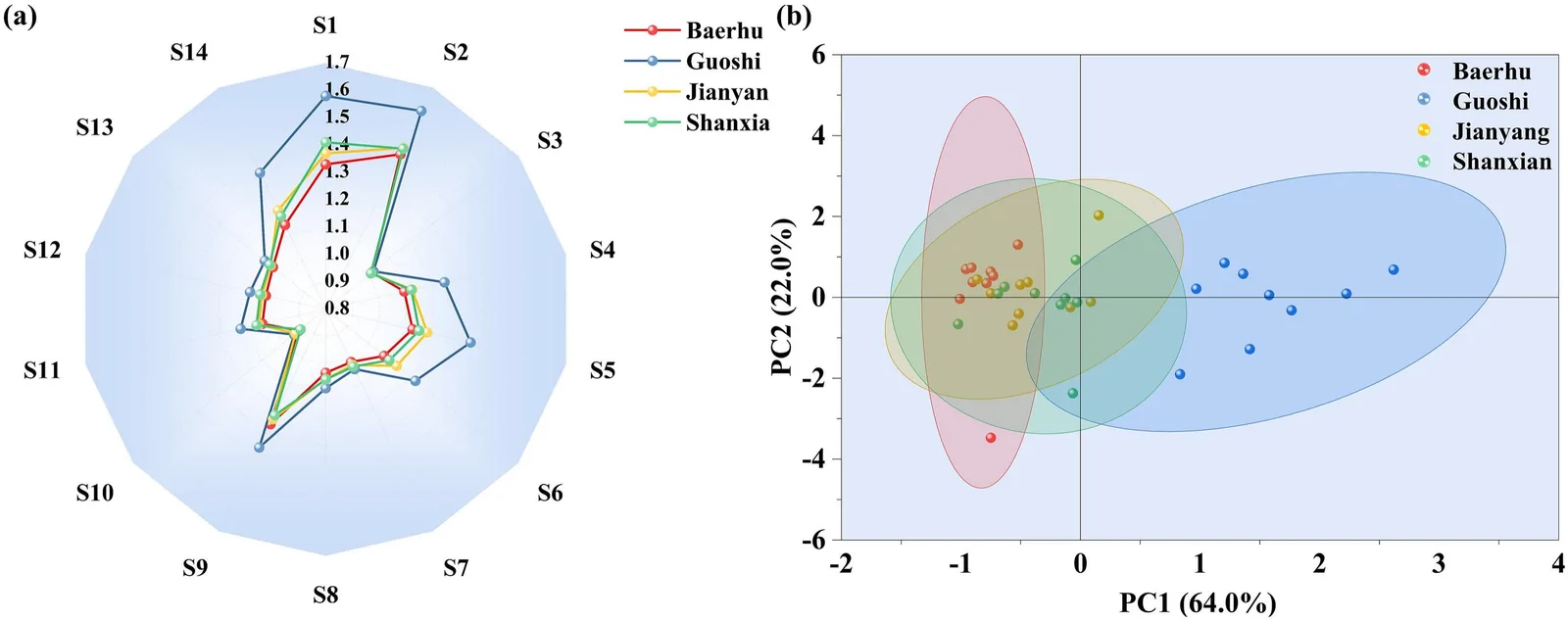
E-nose analysis of four commercial mutton soup products. (a) Radar plot of the mean responses of sensors and (b) PCA biplot based on the sensor responses. For each product, three independently prepared package-level samples were analyzed, with three parallel measurements performed for each sample. Each point in the PCA biplot represents one measurement, giving nine measurements per product.

To further compare the odor characteristics of the samples, PCA was performed based on the response of the E-nose sensors. As shown in Fig. 1b, PC1 and PC2 explained 64.0% and 22.0% of the total variance, respectively, accounting for 86.0% of the cumulative variance. This indicated that the first two principal components retained most of the information contained in the E-nose responses (Leng et al., 2021). Guoshi was located on the positive side of PC1 and was clearly separated from the other products. Most sensors had positive loadings on PC1, indicating that this component represented the overall response intensity of the sensor array. Among them, S4, S11, S12, and S2 made relatively large contributions. These sensors are mainly sensitive to sulfur compounds, allyl sulfides, ketones, alcohols, aldehydes, and short-chain alkanes. Thus, the separation of Guoshi was associated with stronger combined responses to these volatile categories rather than with one specific class of compounds. PC2 was characterized mainly by positive loadings from S10 and S6 and a negative loading from S7. S6 responds to organic gases, anisole, aldehydes, and aromatic compounds, whereas S7 is mainly sensitive to short-chain alkanes, natural gas, and biogas. PC2 therefore reflected a contrast between these sensor response patterns. Baerhu and Jianyang were located close to each other on the negative side of PC1, suggesting relatively similar overall E-nose profiles, whereas Shanxian showed greater variation along PC2. Because metal oxide sensors respond to overlapping groups of volatiles, these results provide only a broad indication of the compound categories associated with sample separation. GC-IMS and GC–MS were subsequently used to identify the specific volatile compounds responsible for these differences.

#### 3.1.2 Volatile fingerprint characterization of four regional mutton soups based on GC-IMS

GC-IMS is a sensitive technique that enables rapid visualization and comparison of volatile profiles without requiring SPME enrichment or sample concentration. Following the E-nose analysis, GC-IMS was therefore used to examine the direct headspace fingerprints of the four mutton soup products and to locate their shared and differential volatile signal regions. Three-dimensional (3D) and two-dimensional (2D) topographic plots are shown in Fig. 2a and b. In these plots, signal intensity is represented by color, with the color gradually changing from blue to red as the concentration of volatile compounds increases (He et al., 2023). Most signals were distributed within the retention time range of 100–700 s. Similar signal patterns were observed among the four samples between 100 and 300 s, indicating the presence of common volatile compounds, although differences in signal intensity were evident. More pronounced differences were observed between 500 and 700 s. Guoshi exhibited a greater number of signal peaks and stronger signal intensities, followed by Jianyang, suggesting a more complex volatile profile. This observation was consistent with the E-nose results, in which Guoshi was clearly separated from the other samples and exhibited a distinctive overall odor profile.

**Fig. 2:**
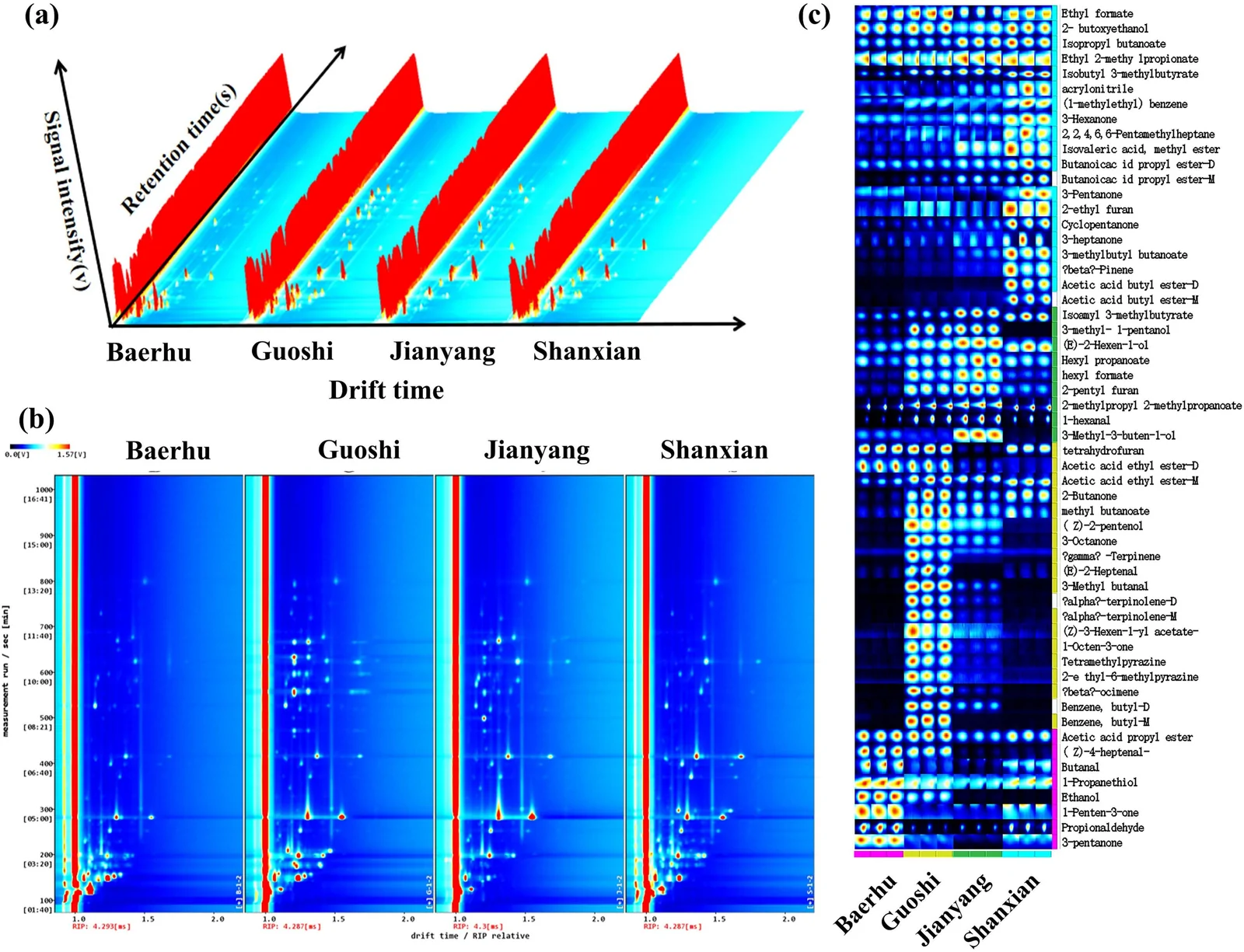
GC-IMS analysis of four mutton soup products. (a) Three-dimensional topographic plots, (b) two-dimensional topographic plots, and (c) volatile fingerprint plot.

A fingerprint plot was subsequently constructed to examine the differences in individual signals (Fig. 2c). A total of 55 signal peaks were detected, and 50 volatile compounds were successfully identified, including 5 aldehydes, 5 alkenes, 7 alcohols, 9 ketones, 16 esters, and 8 other compounds. Baerhu showed relatively strong signals for eight compounds, namely 3-pentanone, (*Z*)-4-heptenal, butanal, 1-propanethiol, ethanol, ethyl acetate, 1-penten-3-one, and propionaldehyde. Guoshi was characterized by 19 relatively strong signals, the largest number among the four samples. Nine compounds were found at relatively strong signals in Jianyang, whereas 16 were more prominent in Shanxian. Ethyl formate, 2-butoxyethanol, isopropyl butyrate, and ethyl 2-methylpropanoate showed relatively strong signals in all four products and formed part of their shared volatile fingerprint. These findings indicated that GC-IMS could distinguish the four products based on their shared and product-associated headspace fingerprints, with Guoshi displaying the most distinct signal pattern. Although GC-IMS provided detailed volatile fingerprints, five of the 55 detected signals remained unidentified. In addition, quantitative analysis by GC-IMS is challenging, and comparisons are generally based on relative peak intensities. Therefore, HS-SPME-GC–MS was subsequently performed to further characterize differences in the composition and relative levels of volatile compounds among the four mutton soup products.

#### 3.1.3 Volatile compounds and potentially odor-active compounds identified by GC–MS analysis

As shown in Table S3, a total of 77 volatile compounds were identified in the four mutton soups by GC–MS, including 15 aldehydes, 12 ketones, 15 alcohols, 6 esters, 14 alkanes, 11 alkenes, and 4 other compounds. Twenty-two potentially odor-active compounds (OAV > 1) were identified across the four mutton soups. Because volatile concentrations were estimated using a single internal standard with an assumed relative response factor of 1, the resulting OAVs were interpreted as indicators of potential odor relevance and used only for comparative screening. In addition, odor thresholds determined in water may not accurately reflect odor perception in the complex matrix of mutton soup, where lipids, proteins, salts, and other components can affect the partitioning and release of volatile compounds. Individual OAVs also do not account for additive, synergistic, masking, or suppressive interactions among odorants.

As shown in Table 1, nonanal, heptanal, octanal, (*E*)-2-octenal, and 1-octen-3-ol exhibited OAV values greater than 1 in all samples, indicating that they may contribute to their shared aroma characteristics. These compounds have been widely reported as key odorants in mutton and mutton-derived products and are mainly generated through lipid oxidation during cooking (Li et al., 2023). Aldehydes such as heptanal, octanal, nonanal, and (*E*)-2-octenal are mainly formed from the oxidation of unsaturated fatty acids, including oleic acid, linoleic acid, linolenic acid, and arachidonic acid (Nachtigall et al., 2019). Heptanal contributes fatty aroma notes, whereas octanal and nonanal are associated with fatty, citrus-like, and green odors (Elmore et al., 1999). (*E*)-2-Octenal is recognized as an important contributor to meaty aroma and can be generated from the oxidation of linoleic acid (Qi et al., 2022). In addition, 1-octen-3-ol, a characteristic alcohol with a mushroom-like aroma, is commonly detected in mutton and is produced through the thermal oxidation of linoleic and arachidonic acids during cooking (Li et al., 2023; Wang et al., 2024). These findings suggest that lipid oxidation-associated compounds may contribute to the shared aroma characteristics of the four mutton soup products.

Notably, Guoshi exhibited substantially higher OAVs for β-myrcene, cuminaldehyde, linalool, d-limonene, and phellandrene than the other samples. These compounds are commonly associated with spices and aromatic herbs (Yuan et al., 2023). β-Myrcene, which showed the highest OAV in Guoshi, is a terpene compound frequently reported in black pepper and other spices (Ozkara et al., 2019). Cuminaldehyde is the characteristic aroma compound of cumin (Yao et al., 2024), whereas d-limonene, linalool, and β-myrcene have been identified in Sichuan pepper and related seasonings (Wang et al., 2021). Phellandrene is also recognized as a key aroma component of fennel (Pan et al., 2025). The relatively high abundance of these spice-associated compounds may have contributed to the distinct aroma profile of Guoshi. However, because the individual spices were not analyzed, their association with the product's seasoning formulation remains an interpretation based on previously reported sources of these compounds.

To compare the relative prominence of the major odorant groups in Guoshi, the OAV of each compound was expressed as a percentage of the summed OAV of all compounds with values greater than 1. The five lipid oxidation-associated aldehydes and alcohols shared by all four products accounted for 34.86% of the total. By comparison, the Guoshi-enriched terpenoids β-myrcene, linalool, d-limonene, and phellandrene accounted for 60.24%. When cuminaldehyde, a spice-associated aromatic aldehyde, was included, the broader spice-associated group accounted for 61.41%. These results indicate that spice-associated odorants were prominent in the estimated odor-activity profile of Guoshi and may have contributed to its separation in the E-nose and GC-IMS analyses.

Collectively, the 22 compounds with OAVs greater than 1 represented several odor qualities, including fatty, green, meaty, mushroom-like, citrus-like, floral, herbal, and spicy notes. However, their relative contributions differed considerably among the four products. The five shared lipid oxidation-associated aldehydes and alcohols accounted for 81.45%, 34.86%, 47.17%, and 86.01% of the summed OAVs in Baerhu, Guoshi, Jianyang, and Shanxian, respectively. Thus, fatty, green, meaty, and mushroom-like notes associated with lipid oxidation appeared to dominate the odor-activity profiles of Baerhu and Shanxian. Jianyang showed a more balanced profile, with contributions from the shared lipid oxidation-associated compounds, 1-hepten-3-one, and β-myrcene. In contrast, the spice-associated group accounted for 61.41% of the summed OAVs in Guoshi, mainly because of the high contribution of β-myrcene, followed by linalool. This result indicates a greater prominence of herbal, spicy, floral, and citrus-like notes in Guoshi and is consistent with its separation in the E-nose and GC-IMS analyses. Overall, the differences among the four products appeared to arise from changes in the relative balance of several odorant groups rather than from the presence of a single compound. These percentages describe the relative distribution of the summed OAVs and do not account for possible synergistic, additive, or masking interactions among odorants.

#### 3.1.4 PLS–DA screening of potential discriminant volatile compounds

PLS–DA was used to examine the separation of the four products and identify volatile compounds associated with their discrimination (Zhang et al., 2023). Twenty-two potentially odor-active compounds (OAV > 1) were included in the model (Fig. 3a). The model yielded R^2^X, R^2^Y, and Q^2^ values of 0.988, 0.998, and 0.995, respectively, indicating a good fit and high internal cross-validation performance within the present dataset. As shown in the score plot, Baerhu, Jianyang, and Shanxian were distributed relatively close to each other, whereas Guoshi was clearly separated from the other samples. This result was consistent with the E-nose, GC-IMS, and OAV analyses, which indicated a distinctive aroma profile for Guoshi. In addition, the loading plot revealed that different volatile compounds contributed to the discrimination of the four samples (Fig. 3b). In the 200-permutation test, the R^2^ and Q^2^ regression-line intercepts were 0.0516 and −0.566, respectively, and the original model had higher R^2^ and Q^2^ values than the permuted models (Fig. 3c). These results indicated that the model showed no evident overfitting (Meng et al., 2021). Given the limited sample size, the model was used for exploratory discrimination and variable screening rather than external prediction.

**Fig. 3:**
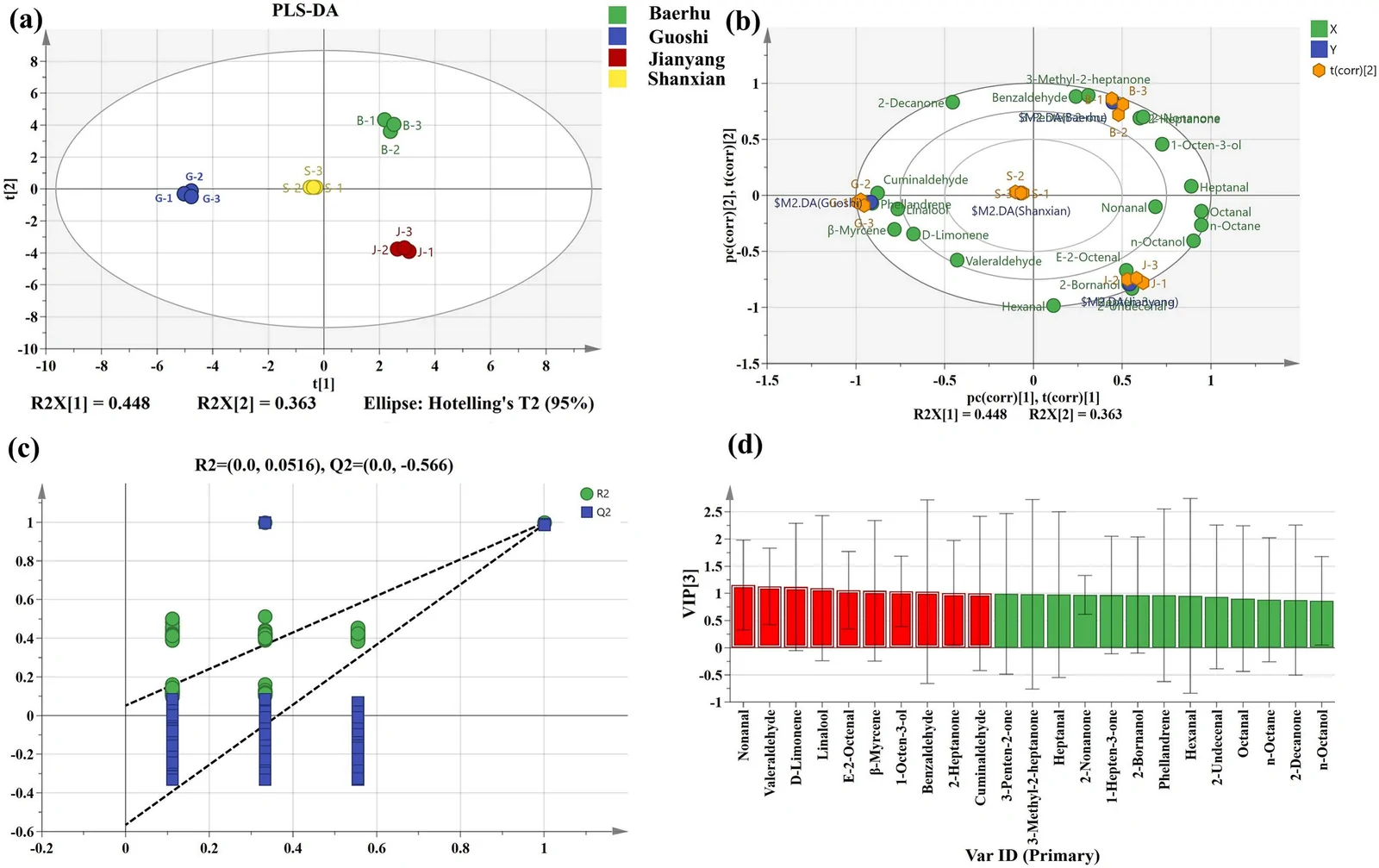
PLS–DA of potentially odor-active compounds with OAVs greater than 1 in four commercial mutton soup products. (a) Score plot, (b) biplot, (c) results of the 200-permutation test, and (d) VIP plot. Each point in the score plot represents one independently prepared package-level sample (*n* = 3 per product). The ellipse represents the 95% Hotelling's T^2^ confidence region. Red bars in the VIP plot indicate compounds with VIP values greater than 1. (For interpretation of the references to color in this figure legend, the reader is referred to the web version of this article.)

Variable importance in projection (VIP) analysis was used to identify the volatile compounds contributing to sample discrimination. Compounds with VIP values greater than 1 are generally considered important discriminant variables (Wang et al., 2023). As shown in Fig. 3d, ten compounds exhibited VIP values above 1, including nonanal, valeraldehyde, d-limonene, linalool, (*E*)-2-octenal, β-myrcene, 1-octen-3-ol, benzaldehyde, 2-heptanone, and cuminaldehyde. Based on their relative levels and positions in the loading plot (Fig. 3b), these compounds were assigned to the product with which they showed the strongest association. Specifically, Baerhu was characterized by 1-octen-3-ol (mushroom-like), benzaldehyde (almond-like), and 2-heptanone (fruity and cheesy). The characteristic compounds associated with Guoshi were d-limonene (citrus-like), linalool (floral and woody), β-myrcene (herbal, balsamic, and pepper-like), and cuminaldehyde (cumin-like and spicy). Jianyang was mainly associated with nonanal, characterized by fatty, green, and citrus-like notes, and (*E*)-2-octenal, which contributes fatty, green, and meaty notes. Valeraldehyde, which has a fruity, malty, and pungent odor, showed the strongest association with Shanxian. Thus, the ten VIP-selected compounds were classified into three Baerhu-associated, four Guoshi-associated, two Jianyang-associated, and one Shanxian-associated potential aroma markers.

Overall, the product differentiation was associated with variations in volatile compounds. However, because the compounds were selected using multivariate statistical analysis and semi-quantitative volatile data, they should be regarded as potential discriminant aroma markers rather than definitive chemical markers of the corresponding regions.

### 3.2 Taste characteristics of four mutton soups

#### 3.2.1 Overall taste profiles and sample discrimination based on *E*-tongue responses

The E-tongue simulates the human gustatory system through electronic sensors and is widely used to evaluate and distinguish taste characteristics of foods (Guan et al., 2025). In the present study, E-tongue analysis was employed to obtain an overall understanding of the taste profiles of the four mutton soups. As shown in Fig. 4a, all samples exhibited relatively high response values for the umami and saltiness sensors, indicating that these two taste attributes were dominant in all soups. Although differences in the intensities of individual taste attributes were observed, the radar plots showed considerable overlap among the four samples, suggesting similarities in their overall taste profiles (Gervis et al., 2021). Notably, Shanxian and Jianyang exhibited relatively higher responses for saltiness than the other samples.

**Fig. 4:**
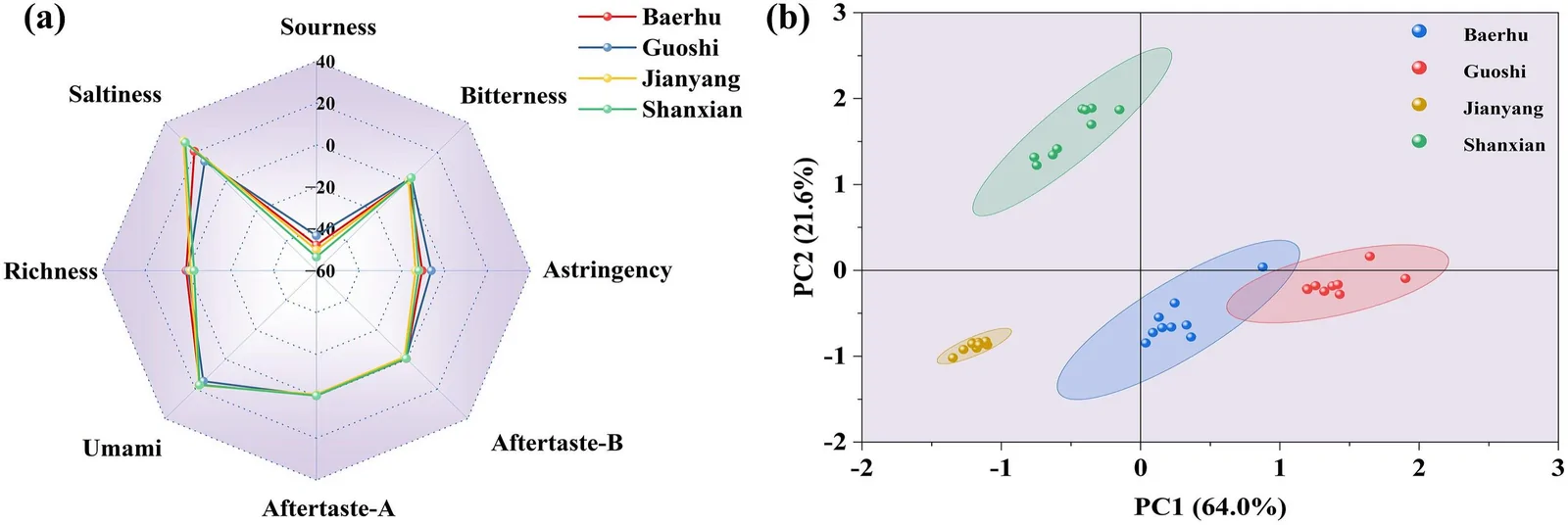
E-tongue analysis of four commercial mutton soup products. (a) Radar plot of the mean responses of sensors and (b) PCA biplot based on the sensor responses. For each product, three independently prepared package-level samples were analyzed, with three parallel measurements performed for each sample. Each point in the PCA biplot represents one measurement, giving nine measurements per product.

To further discriminate the taste characteristics of the samples, PCA was performed based on the E-tongue data. As shown in Fig. 4b, PC1 and PC2 explained 64.0% and 21.6% of the total variance, respectively, accounting for 85.6% of the cumulative variance. This result indicates that the PCA model adequately represented the taste information of the samples. Baerhu and Guoshi were located relatively close to each other in the score plot, suggesting similarities in their taste profiles. In contrast, Shanxian and Jianyang were distributed in distinct regions, indicating noticeable differences from Baerhu and Guoshi. These results suggest that, although the four mutton soups shared similar dominant taste attributes, variations in the composition and abundance of taste-active compounds contributed to their distinguishable taste profiles. Therefore, the major taste-active compounds, including free amino acids and 5′-nucleotides, were further analyzed to elucidate the chemical basis underlying these taste differences.

#### 3.2.2 Free amino acid composition and taste activity analysis

FAAs are important taste-active compounds that contribute significantly to the overall taste characteristics of foods (Adcock et al., 2016). Based on their taste attributes, the detected FAAs in the four mutton soups were classified into umami, sweet, and bitter amino acids (Table 2). Sweet amino acids represented the largest proportion of total FAAs in all samples, with proline being the most abundant amino acid, followed by the umami amino acid glutamic acid. Bitter amino acids were present at relatively lower concentrations. However, the contribution of an amino acid to taste depends not only on its concentration but also on its taste threshold. Therefore, its potential contribution was assessed using the TAV. A TAV greater than 1 indicates that the compound may be perceptible individually, whereas a value below 1 suggests that its direct taste contribution is likely to be limited, although interactions with other taste compounds may still occur (Xian et al., 2025). Despite its high concentration, proline had a relatively low TAV because of its high taste threshold. In contrast, glutamic acid exhibited a higher TAV relative to its concentration, indicating greater potential relevance to taste perception. Umami amino acids, particularly glutamic acid and aspartic acid, are recognized responsible for the umami taste of meat products, whereas sweet amino acids can improve palatability and may synergistically enhance umami perception (Lai et al., 2022).

**Table 2:** Concentrations of FAAs and 5′-nucleotides in four commercial mutton soup products.

Taste-active compounds	Concentration (mg/L)				Taste threshold (mg/L)	TAV			
	Baerhu	Guoshi	Jianyang	Shanxian		Baerhu	Guoshi	Jianyang	Shanxian
Aspartic acid	4.52 ± 0.67^b^	8.97 ± 0.45^a^	3.18 ± 0.25^c^	0.58 ± 0.07^d^	1000	<0.01	0.01	<0.01	<0.01
Glutamic acid	175.21 ± 2.16^c^	397.09 ± 2.62^b^	789.03 ± 6.53^a^	127.52 ± 1.39^d^	300	0.58	1.32	2.63	0.43
**Σ** Umami amino acid	179.73 ± 2.26^c^	406.07 ± 2.66^b^	792.21 ± 6.53^a^	128.1 ± 1.39^d^					
Threonine	5.97 ± 2.36^b^	10.23 ± 0.24^a^	5.31 ± 0.26^b^	0.84 ± 0.01^c^	2600	<0.01	<0.01	<0.01	<0.01
Serine	21.9 ± 3.22^a^	18.73 ± 0.23^b^	10.73 ± 0.09^c^	1.23 ± 0.1^d^	1500	0.01	0.01	0.01	<0.01
Glycine	8.76 ± 0.19^c^	41.58 ± 0.58^a^	17.35 ± 0.5^b^	1.76 ± 0.03^d^	1300	0.01	0.03	0.01	<0.01
Proline	146.15 ± 3.02^c^	354.03 ± 1.09^b^	1186.3 ± 5.26^a^	126.47 ± 3.08^d^	3000	0.05	0.12	0.40	0.04
Alanine	37.22 ± 1.15^a^	30.96 ± 1.9^b^	23.96 ± 0.59^c^	3.35 ± 0.05^d^	600	0.06	0.05	0.04	0.01
**Σ** Sweet amino acid	219.99 ± 4.93^c^	455.53 ± 2.22^b^	1243.65 ± 5.29^a^	133.65 ± 3.08^d^					
Valine	9.39 ± 0.46^b^	13.31 ± 0.19^a^	8.86 ± 0.24^b^	1.94 ± 0.18^c^	400	0.02	0.03	0.02	<0.01
Methionine	12.56 ± 1.26^a^	14.65 ± 2.96^a^	12.13 ± 0.45^a^	3.01 ± 0.1^b^	300	0.04	0.05	0.04	0.01
Isoleucine	12.04 ± 2.84^b^	16.4 ± 1.08^a^	9.27 ± 0.13^b^	1.71 ± 0.05^c^	900	0.01	0.02	0.01	<0.01
Leucine	18.54 ± 1.01^b^	32.13 ± 9.34^a^	16.79 ± 0.5^b^	2.66 ± 0.59^c^	1900	0.01	0.02	0.01	<0.01
Tyrosine	7.62 ± 2.87^b^	11.91 ± 5.84^a^	7.42 ± 0.34^b^	1.24 ± 0.03^c^	ND	ND	ND	ND	ND
Phenylalanine	4.57 ± 2.18^b^	17.27 ± 5.29^a^	1.69 ± 0.26^b^	0.53 ± 0.31^b^	900	0.01	0.02	<0.01	<0.01
Histidine	29.89 ± 0.67^a^	22.44 ± 3.28^b^	30.42 ± 0.33^a^	6.5 ± 0.05^c^	200	0.15	0.11	0.15	0.03
Lysine	20.28 ± 0.99^a^	20.59 ± 2.08^a^	11.88 ± 0.18^b^	2.5 ± 0.04^c^	500	0.04	0.04	0.02	0.01
Arginine	30.9 ± 2.54^a^	11.69 ± 1.51^b^	11.07 ± 0.61^b^	2.19 ± 0.28^c^	500	0.06	0.02	0.02	<0.01
**Σ** Bitter amino acid	145.79 ± 5.63^b^	160.39 ± 13.29^a^	109.53 ± 1.11^c^	22.28 ± 0.76^d^					
**Σ** Total amino acid	545.01 ± 6.65^c^	1001.08 ± 11.99^b^	2125.4 ± 8.39^a^	280.07 ± 3.38^d^					
5′-AMP	15.07 ± 0.49^b^	11.28 ± 0.69^c^	42.38 ± 0.84^a^	6.37 ± 0.09^d^	500	0.03	0.02	0.08	0.01
5′-GMP	23.55 ± 0.04^c^	29.41 ± 0.27^b^	15.39 ± 1.9^d^	29.76 ± 0.15^a^	125	0.19	0.24	0.12	0.24
5′-IMP	11.59 ± 0.2^a^	6.86 ± 0.04^c^	9.12 ± 0.57^b^	5.79 ± 0.08^d^	250	0.05	0.03	0.04	0.02
**Σ** 5′ -Nucleotides	50.21 ± 0.53^b^	47.55 ± 0.74^b^	66.89 ± 2.15^a^	41.92 ± 0.19^c^					

ND, not detected. Different superscript letters within the same row indicate significant differences among samples (*P* < 0.05).

Significant differences in FAA composition and taste activity were observed among the four samples. Jianyang exhibited the highest TAV for glutamic acid (2.63), followed by Guoshi (1.32), whereas considerably lower values were found in Baerhu and Shanxian. Given the important role of glutamic acid in umami perception, the higher TAV values in Jianyang and Guoshi may contribute to their stronger umami characteristics. Among the bitter amino acids, histidine showed the highest TAV, the TAVs of all bitter amino acids were below 1, indicating that their individual contributions to bitterness were likely limited. Although all four products underwent the same microwave reheating procedure before analysis, their thermal histories during commercial production were unknown. The FAA composition of cooked meat broth reflects the release and transfer of amino acids from meat proteins into the aqueous phase during heating, while some amino acids may also be consumed through thermal reactions (Li & Liu, 2022; Su et al., 2025). Differences in the original cooking temperature and duration may therefore have contributed to the variation in FAA composition among the products. In addition, although the FAA profiles of the sheep breeds used in these four products have not been compared under the same conditions, breed has been reported to influence the amino acid composition of mutton (Xiang et al., 2023). Differences in raw materials may thus provide another possible explanation for the observed variation. However, the effects of sheep breed could not be separated from those of ingredient composition and commercial processing in the present study and require further verification.

Overall, differences in FAA composition and taste activity may partly explain the variation in the *E*-tongue responses. However, FAAs alone cannot fully account for the taste characteristics of the soups. Taste-active nucleotides were therefore subsequently determined to provide a more complete interpretation.

#### 3.2.3 5′-nucleotide composition analysis

Nucleotides are important taste-active compounds formed during the degradation of nucleic acids during food processing (Xia et al., 2025). Together with free amino acids, they contribute significantly to the taste characteristics of meat products through synergistic interactions (Yang et al., 2025). Among the detected nucleotides, 5′-AMP is mainly associated with sweetness, whereas 5′-IMP and 5′-GMP are recognized as key contributors to umami taste (Mottram & Nobrega, 2002). As shown in Table 2, the nucleotide compositions differed among the four mutton soups. Jianyang contained the highest concentration of 5′-AMP (42.38 ± 0.84 mg/L), suggesting a greater contribution from sweetness-related taste compounds. In contrast, the combined concentrations of 5′-IMP and 5′-GMP were comparable among Baerhu, Guoshi, and Shanxian and slightly higher than those in Jianyang. However, Jianyang exhibited the highest total concentration of 5′-nucleotides among all samples. Since the sensory contribution of nucleotides depends not only on their individual concentrations but also on their synergistic interactions with umami amino acids, differences in nucleotide composition may influence the overall taste characteristics of the soups. To further evaluate the combined contribution of free amino acids and 5′-nucleotides to umami perception, the EUC was subsequently calculated.

#### 3.2.4 Evaluation of umami intensity based on EUC

EUC, which reflects the synergistic effect between umami amino acids and 5′-nucleotides, is widely used to evaluate the overall umami intensity of foods. Jianyang exhibited the highest EUC value (0.58 g MSG/100 mL), followed closely by Guoshi (0.41 g MSG/100 mL), whereas substantially lower values were observed in Baerhu (0.16 g MSG/100 mL) and Shanxian (0.13 g MSG/100 mL). Notably, the EUC values of Jianyang and Guoshi were approximately three times higher than those of Baerhu and Shanxian, indicating a markedly stronger umami intensity. The higher EUC values of Jianyang and Guoshi may reflect differences in raw materials and processing conditions. First, sheep breed and muscle composition can affect the initial concentrations of free glutamic acid and other taste-active compounds in meat, which may subsequently influence their levels in the cooked broth (Ma et al., 2023). However, the initial FAA composition of raw meat was not determined in the present study, and a direct breed effect therefore cannot be established. Second, the concentration of nucleotides is affected by both heating time and temperature. Previous studies have reported an increase in IMP concentration with prolonged stewing, which may be related to the degradation of ATP-related precursors and the transfer of IMP from meat into the broth. At the same time, continued heating can promote the thermal degradation of IMP to inosine (Wang et al., 2026). The final IMP concentration therefore reflects the balance between its formation, release, and degradation during cooking, and differences in thermal processing may contribute to the variation observed among the products. (Huang et al., 2025). Third, seasoning formulations may affect the transfer of FAAs from meat into the aqueous phase during cooking. Ingredients such as salt and certain spices have been reported to modify mass transfer and the release of taste compounds in meat broths (Wang et al., 2020). Nevertheless, because the detailed spice formulations and processing parameters of the commercial products were not disclosed, their specific effects could not be determined. Overall, the higher EUC values of Jianyang and Guoshi reflected the combined concentrations of umami amino acids and 5′-nucleotides and their calculated synergistic effects. However, differences in EUC among the products should be interpreted as the combined outcome of raw-material composition, seasoning formulation, and processing conditions rather than being attributed to any single factor.

### 3.3 Sensory evaluation and correlation with instrumental analyses

The sensory evaluation results are shown in Fig. S1. Jianyang received the highest scores for taste, aroma, and overall acceptability, followed by Baerhu, whereas Guoshi received the lowest aroma score. Although Jianyang had both the highest sweet FAA content and the highest taste and overall acceptability scores, Guoshi contained more sweet FAAs than Baerhu but received lower sensory scores. The correspondence between total sweet FAA content and sensory performance was therefore only partial, indicating that sweet FAA content alone could not explain the differences in palatability among the products. Notably, Guoshi showed the most distinct volatile fingerprint in the E-nose and GC-IMS analyses and had a relatively high proportion of spice-associated volatile compounds, but these characteristics were not accompanied by higher sensory scores. This finding indicates that the diversity or signal intensity of volatile compounds does not necessarily correspond to sensory acceptability. A balanced aroma profile may be more important than volatile complexity. Jianyang also had the highest EUC and taste score, suggesting that the synergistic effect of umami amino acids and 5′-nucleotides may be associated with its favorable taste. Guoshi, however, had a similarly high EUC but lower sensory scores than Jianyang, indicating that EUC reflects potential umami intensity but cannot independently explain overall acceptability.

To further examine the relationship between flavor composition and sensory performance, correlation analysis was conducted between the sensory attributes and the OAVs of volatile compounds, the TAVs of FAAs and nucleotides, and EUC (Fig. 5). Among the volatile compounds, octanal, nonanal, (*E*)-2-octenal, 2-undecenal, 1-hepten-3-one, and 1-octanol were generally positively correlated with overall acceptability. These compounds are associated with fatty, green, or meaty odors and may be related to a more balanced aroma background in mutton soup. In contrast, cuminaldehyde, 2-decanone, d-limonene, phellandrene, and β-myrcene were generally negatively correlated with overall acceptability. Cuminaldehyde and several terpenes also showed relatively strong negative correlations with the aroma score. Considered together with the OAV profile of Guoshi, these results suggest that the prominence of spicy, herbal, and citrus-like notes may have increased its aroma distinctiveness without necessarily improving aroma balance or sensory acceptability. Similarly, negative correlations of limonene and β-phellandrene with overall acceptability have been reported in a seasoned soup system, suggesting that pronounced spice-associated volatile profiles may not necessarily contribute positively to sensory acceptance (Yan et al., 2025).

**Fig. 5:**
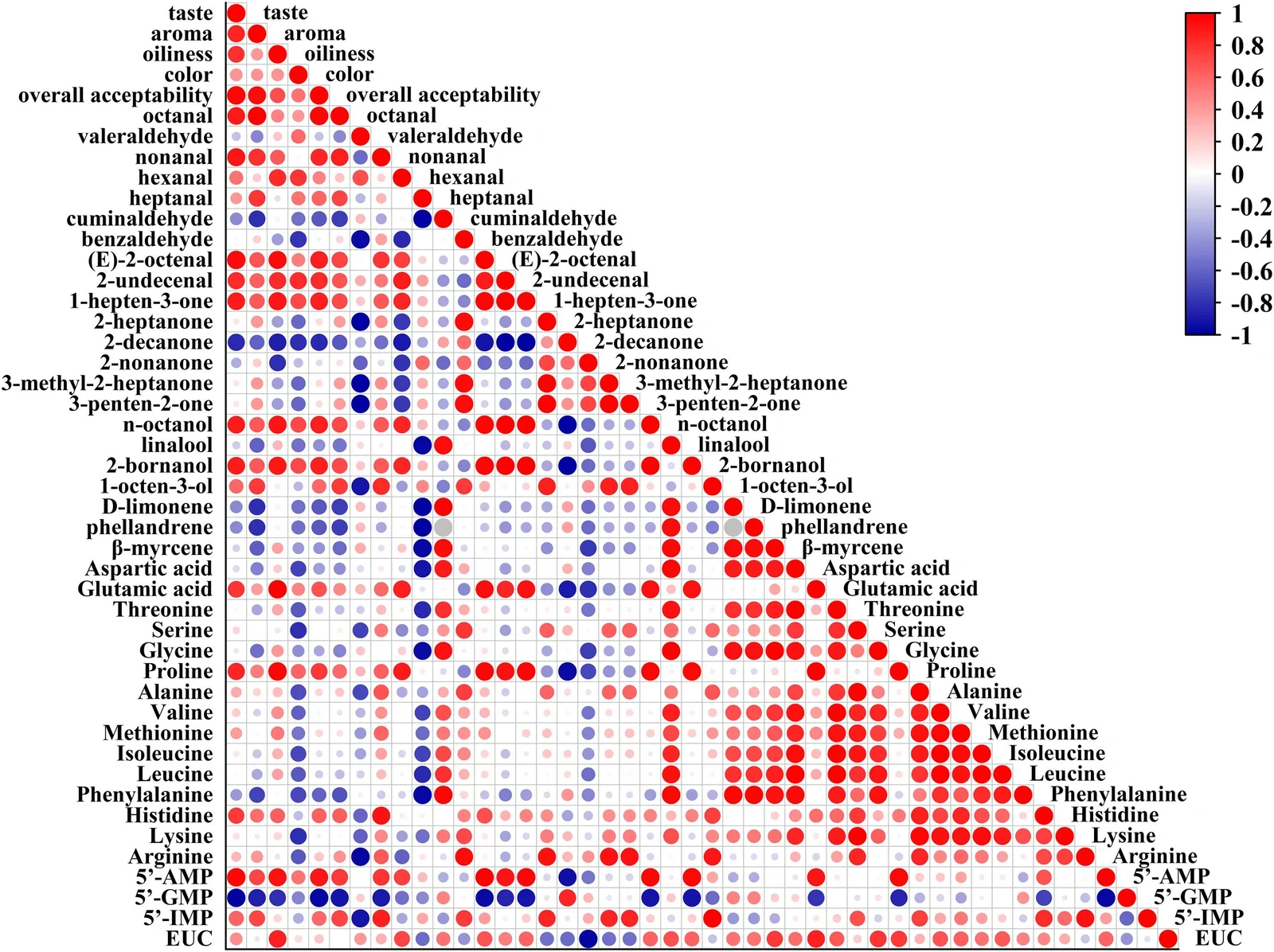
Person correlation matrix between sensory attributes and flavor-related indices of the four commercial mutton soup products. The analysis included the estimated OAVs of volatile compounds, TAVs of free amino acids and 5′-nucleotides, and EUC. Red and blue circles indicate positive and negative correlations, respectively, while circle size and color intensity represent the absolute magnitude of the correlation coefficient. Correlations were calculated using the product-level mean values of the four products. (For interpretation of the references to color in this figure legend, the reader is referred to the web version of this article.)

Among the non-volatile compounds, glutamic acid, proline, and 5′-AMP were generally positively correlated with the taste score, while glutamic acid and proline were also positively correlated with overall acceptability. This finding is consistent with a previous study on beef, in which glutamic acid and proline were significantly and positively correlated with overall acceptability (Piao et al., 2015). EUC was positively correlated with the taste score, although the association was moderate, and showed only a weak positive correlation with overall acceptability. Thus, the synergy between umami amino acids and 5′-nucleotides appears to contribute to taste but does not fully account for the overall sensory performance of the products, which is also influenced by aroma, saltiness, oiliness, color, and interactions among flavor components. Although 5′-GMP was negatively correlated with most sensory attributes, this pattern may reflect its distribution across the four products and should not be interpreted as evidence that 5′-GMP adversely affects sensory quality. Overall, the sensory differences among the products appeared to be more closely related to the balance between volatile and non-volatile flavor components than to any single compound or group of taste-active compounds. Because only four commercial products were compared, the correlation analysis was used to identify patterns of association and does not establish causal relationships.

## 4 Conclusion

This study compared the aroma and taste characteristics of four commercial mutton soups from different regions in China. Clear differences were observed in their aroma and taste profiles. Lipid oxidation-associated compounds with estimated OAVs greater than 1 were common to all four products and may contribute to their shared aroma characteristics. Guoshi showed the most distinct volatile profile and contained relatively high levels of several spice-associated compounds, although their specific origins could not be confirmed. PLS–DA identified ten potential discriminant volatile compounds. Jianyang and Guoshi had higher EUC values than Baerhu and Shanxian, reflecting greater calculated synergy between umami amino acids and 5′-nucleotides. Jianyang also received the highest taste, aroma, and overall acceptability scores, whereas the distinct and complex volatile profile of Guoshi was not accompanied by higher sensory scores. These findings suggest that high sensory score may be more closely associated with the balance between aroma and taste than with volatile complexity or calculated umami intensity alone. This study was limited to four finished commercial products, with one product representing each region. The results therefore cannot be generalized to all mutton soups from the corresponding regions. In addition, flavor development during stewing was not monitored, and consumer preference was not evaluated in a large-scale sensory test. Future studies should include products from multiple manufacturers and production batches, track changes in flavor compounds during stewing, and conduct broader consumer testing. Such work may support flavor regulation in instant mutton soup and the development of seasoning formulations that reduce gamey notes while maintaining a balanced umami taste.

## CRediT authorship contribution statement

**Can Xiang:** Writing – review & editing, Writing – original draft, Visualization, Data curation. **Xue Dai:** Writing – review & editing, Writing – original draft, Visualization, Investigation. **Zhaojun Chen:** Writing – review & editing, Supervision, Methodology. **Chunjiang Zhang:** Writing – review & editing, Supervision, Resources, Methodology. **Dequan Zhang:** Writing – review & editing, Supervision, Resources, Conceptualization. **Sining Li:** Writing – review & editing, Supervision, Resources, Conceptualization. **Caiyan Huang:** Writing – review & editing, Supervision, Resources, Methodology, Conceptualization.

## Declaration of competing interest

The authors declare that they have no known competing financial interests or personal relationships that could have appeared to influence the work reported in this paper.

## Data Availability

Data will be made available on request.
