# Supplementary material for: Integrated characterization of aroma, taste, and sensory properties of four commercial mutton soups from different regions of China
Source: Food Chem X. 2026 Oct 1;39:104559. doi: 10.1016/j.fochx.2026.104559 (PMC13660535; doi:10.1016/j.fochx.2026.104559)
Supplement: Supplementary file 1 [file mmc1.docx]

Table S1. Electronic nose sensors and corresponding sensitive substances

| No. | Sensor | Sensitive substances |
| --- | --- | --- |
| 1 | S1 | Propane and fumes |
| 2 | S2 | Short-chain alkanes, alcohols, and aldehydes |
| 3 | S3 | Ozone |
| 4 | S4 | Sulfur compounds, including hydrogen sulfide |
| 5 | S5 | Nitrogen-containing compounds, including ammonia |
| 6 | S6 | Aldehydes, ethers, and aromatic compounds |
| 7 | S7 | Short-chain alkanes, methane, and natural gas |
| 8 | S8 | Liquefied gas |
| 9 | S9 | Short-chain alkanes, petroleum ether odor |
| 10 | S10 | Hydrogen |
| 11 | S11 | Allyl sulfide |
| 12 | S12 | Ketones and alcohols |
| 13 | S13 | Methane |
| 14 | S14 | Combustible gases |

Table S2. Sensory evaluation protocols

| Items | Evaluation standards | | | |
| --- | --- | --- | --- | --- |
|  | Excellent (10-8) | Good (7-5) | Fair (4-2) | Poor (1-0) |
| Taste | Smooth mouthfeel, pronounced umami, rich meatiness, prolonged aftertaste, and well-balanced salinity | Pure taste, mild umami, subtle meatiness, fleeting aftertaste, and leaning towards salty/bland | Flat flavor, weak umami, muted meatiness, and overly salty/insipid | Fairly ordinary taste and lacking in umami |
| Aroma | Strong aroma, no gaminess | Fairly strong aroma, no gaminess | Fairly strong aroma, slight gaminess | Barely any aroma, pronounced gaminess |
| Oily | No noticeable floating oil and homogeneous | Minor oil particles without cloudiness | Small patches of oil with slight cloudiness | Large patches of thick floating oil with obvious cloudiness |
| Color | Milky white | Yellow | Pale yellow | Colorless |
| Overall Acceptability | Very high overall acceptability | High overall acceptability | Moderate overall acceptability | Low overall acceptability |

Table S3. The concentrations of volatile compounds in four commercial mutton soups

| Category | Compounds | | Concentration (μg/L) | | | |
| --- | --- | --- | --- | --- | --- | --- |
|  |  |  | Baerhu | Guoshi | Jianyang | Shanxian |
| Aldehydes | | Octanal | 81.21±3.59^b^ | 26.97±0.75^d^ | 96.16±6.3^a^ | 47±3.06^c^ |
|  |  | Valeraldehyde | 9.71±0.92^c^ | 14.28±0.90^b^ | 13.71±0.23^b^ | 16.53±0.38^a^ |
|  |  | Nonanal | 183.46±2.53^b^ | 111.98±1.56^c^ | 200.87±5.42^a^ | 73.23±2.27^d^ |
|  |  | Hexanal | - | 44.89±2.22^b^ | 98.02±8.01^a^ | 43.39±1.46^b^ |
|  |  | Heptanal | 83.76±4.16^a^ | 26.73±0.16^b^ | 82.52±2.68^a^ | 85.84±2.68^a^ |
|  |  | Decanal | 13.74±0.65^a^ | 4.29±0.02^b^ | 2.38±0.50^c^ | 4.17±0.49^b^ |
|  |  | Cuminaldehyde | 26.59±2.53^b^ | 194.16±6.93^a^ | - | - |
|  |  | Cinnamaldehyde | 7.5±0.46 | - | - | - |
|  |  | Piperonal | - | 7.58±0.05^a^ | 1.16±0.04^b^ | - |
|  |  | Benzaldehyde | 103.26±4.43^a^ | 31.26±0.49^b^ | 8.7±0.54^c^ | 12.81±0.14^c^ |
|  |  | (*E*)-2-Octenal | 12.83±1.32^b^ | 11.44±1.21^b^ | 35.42±0.94^a^ | 4.75±0.29^c^ |
|  |  | 4-Methoxybenzaldehyde | 14.54±1.37 | - | - | - |
|  |  | 3-Isopropylbenzaldehyde | 21.97±1.31^a^ | - | 1.98±1.06^b^ | - |
|  |  | 2-Undecenal | - | - | 18.91±0.44^a^ | 3.76±0.03^b^ |
|  |  | (*E*)-3,7-Dimethyl-2,6-octadienal | 24.94±0.34^a^ | - | 15.61±0.45^b^ | - |
| Ketones | | 1,3,3-Trimethylbicyclo[2.2.1]heptan-2-one | 39.64±4.06 | - | - | - |
|  |  | 1-Hepten-3-one |  |  | 7.14±0.35 |  |
|  |  | 2-Heptanone | 7.2±0.17^a^ | - | 1.65±0.54^b^ | - |
|  |  | 2-Decanone | 6.06±1.04^a^ | 5.76±0.60^a^ | 2.8±0.09^b^ | 5.08±0.03^a^ |
|  |  | 2-Nonanone | 8.5±0.39^a^ | 1.73±0.66^d^ | 3.64±0.14^c^ | 6.44±0.24^b^ |
|  |  | 3-Methyl-2-heptanone | 19.05±2.77^a^ | 3.18±0.14^b^ | - | 2.08±0.03^b^ |
|  |  | 3-Hydroxy-2-butanone | 439.24±66.24^a^ | 3.24±0.44^b^ | 8.09±1.83^b^ | 3.39±0.06^b^ |
|  |  | 3-Penten-2-one | 43.49±1.74 | - | - | - |
|  |  | Menthone | - | 10.26±0.24^a^ | - | 0.34±0.07^c^ |
|  |  | Acetone | 95.55±3.71^a^ | 2.28±0.36^b^ | - | 3.85±0.29^b^ |
|  |  | Cyclopropylmethyl ketone | 61.06±0.54 | - | - | - |
|  |  | Methylheptenone | 43.51±0.22^a^ | 4.9±0.31^c^ | 14.24±1.09^b^ | - |
| Alcohols | | n-Octanol | 50.27±6.85^b^ | 8.08±0.12^c^ | 86.76±11.16^a^ | 45.05±4.06^b^ |
|  |  | n-Pentanol | - | - | 9.77±0.38^a^ | 7.3±0.06^b^ |
|  |  | n-Hexanol | 135.67±2.70^a^ | - | 52.85±6.90^b^ | 26.12±1.80^c^ |
|  |  | n-Heptanol | 74.95±4.64^b^ | 6.54±0.91^d^ | 88.43±1.82^a^ | 40.95±1.48^c^ |
|  |  | Cinnamyl alcohol | 8.39±0.89 |  |  |  |
|  |  | Linalool | 49.65±0.64^c^ | 220.12±6.86^a^ | 63.77±1.01^b^ | 1.75±0.01^d^ |
|  |  | p-Mentha-1,5-dien-8-ol | - | 18.61±1.08 | - | - |
|  |  | Phenethyl alcohol | 85.99±4.60^a^ | 4.67±0.39^b^ | 1.26±0.04^b^ | 0.89±0.08^b^ |
|  |  | Benzyl alcohol | 18.31±0.97^a^ | - | - | 0.76±0.02^b^ |
|  |  | Eucalyptol | 51.92±1.70^a^ | 7.12±1.37^c^ | 28.95±3.37^b^ | - |
|  |  | α-Terpineol | 28.65±0.38^a^ | 27.67±0.44^b^ | 11.06±0.44^c^ | 2.56±0.48^d^ |
|  |  | 2-Bornanol | - | - | 11.78±0.73 | - |
|  |  | 1-Octen-3-ol | 38.04±0.52^a^ | 9.47±0.36^c^ | 23.34±0.21^b^ | 8.28±0.17^d^ |
|  |  | Nonanol | 26.73±4.52^b^ | 4.39±0.38^d^ | 14.21±3.25^c^ | 40.4±2.84^a^ |
|  |  | (-)-Terpinen-4-ol | 53.14±4.00^a^ | - | 4.99±1.01^b^ | - |
| Esters | | Vinyl hexanoate | 113.41±5.86^a^ | 77.57±6.53^b^ | - | 30.52±1.56^c^ |
|  |  | Dibutyl phthalate | 15.36±0.32^a^ | 1.00±0.20^b^ | - | - |
|  |  | 2-Ethylhexyl hexyl sulfite | 7.76±0.98^a^ | 1.48±0.28^b^ | - | - |
|  |  | Isobutyl pentyl sulfite | 12.69±0.62^a^ | 2.38±0.11^b^ | - | - |
|  |  | Linalyl acetate | - | 5.06±0.75^a^ | 25.37±1.53^b^ | - |
|  |  | Bornyl acetate | 88.48±5.43^a^ | - | 3.54±0.85^b^ | - |
| Alkanes | | n-Octane | 28.7±1.36^b^ | 8.03±0.33^d^ | 38.92±2.68^a^ | 18.94±0.90^c^ |
|  |  | n-Undecane | 131.81±11.02^a^ | 4.59±0.02^b^ | 5.22±0.59^b^ | 2.23±0.27^b^ |
|  |  | n-Dodecane | 69.07±4.21^a^ | 7.40±0.37^c^ | 34.30±5.30^b^ | - |
|  |  | 5-Ethyl-2-methyloctane | 482.16±1.80 | - | - | - |
|  |  | 4-Methyldodecane | 84.27±10.33^a^ | 17.98±0.43^b^ | - | - |
|  |  | 4,7-Dimethylundecane | 185.58±4.83^a^ | 14.64±0.09^b^ | - | - |
|  |  | 4,6-Dimethylundecane | 78.11±2.87^a^ | 11.07±0.55^b^ | - | - |
|  |  | 4,6-Dimethyldodecane | 337.26±14.87^a^ | 34.61±1.12^b^ | - | - |
|  |  | 4-Methyldecane | 258.62±9.25^a^ | 12.75±0.62^b^ | - | - |
|  |  | 2-Methylundecane | 93.51±1.99^a^ | 17.4±0.25^b^ | - | - |
|  |  | 2,6-Dimethylundecane | 110.32±2.47^a^ | 25.41±0.18^b^ | - | - |
|  |  | 2,6-Dimethyldecane | 268.91±2.55^a^ | 15.47±1.97^b^ | - | - |
|  |  | 2,6-Dimethylnonane | 223.88±25.74^a^ | 16.22±0.46^b^ | - | - |
|  |  | 2,4-Dimethyldodecane | 163.32±6.96^a^ | 26.23±0.54^b^ | - | - |
| Alkenes | | α-Curcumene | - | 3.32±0.38^b^ | 5.15±0.20^a^ | - |
|  |  | D-Limonene | 117.92±4.69^c^ | 773.76±22.94^a^ | 358.65±13.66^b^ | - |
|  |  | α-Caryophyllene | - | 59.82±11.94^a^ | 10.87±0.62^b^ | - |
|  |  | β-Elemene | - | 27.82±2.66 |  | - |
|  |  | β-Pinene | - | 100.98±7.34^a^ | 25.44±0.86^b^ | - |
|  |  | γ-Terpinene | - | 21.25±3.87^a^ | 4.07±0.24^b^ | - |
|  |  | Sabinene | - | 9.56±0.39^b^ |  | - |
|  |  | Camphene | 12.14±0.30^b^ | 4.82±0.88^c^ | 29.58±5.02^a^ | - |
|  |  | Phellandrene | 2.84±0.20^b^ | 272.21±17.26^a^ | 4.59±2.05^b^ | 0.32±0.16^b^ |
|  |  | Terpinolene | - | 51.02±4.72^a^ | 6.19±1.39^b^ | - |
|  |  | β-Myrcene | 10.93±0.89^c^ | 233.94±6.56^a^ | 74.1±0.64^b^ | - |
| Others | | 2,6-Diethylpyrazine | 19.69±0.35^a^ | - | 0.51±0.09^b^ | - |
|  |  | 2-Ethylfuran | 23.17±1.55^a^ | 1.77±0.04^c^ | 1.73±0.04^c^ | 7.83±0.17^b^ |
|  |  | Caryophyllin | - | 15.27±0.49 | - | - |
|  |  | n-Hexanoic acid | 14.71±1.34^a^ | - | 0.52±0.13^b^ | - |

“-” indicates that the compound was not detected. Different lowercase letters within the same row indicate significant differences among the four products (*P* < 0.05), whereas values sharing the same letter are not significantly different.

Fig. S1. Sensory evaluation profiles of four mutton soups
